# Case of Sensitive Dentine

**Published:** 1859-07

**Authors:** J. Taft


### CASE OF SENSITIVE DENTINE.

BY J. TAFT.

DEC. 24th, 1858.—Mr. L., aged 30, bilious-sanguine temperament, called to have two superior molars filled; the second superior molar of the left side, with a crown cavity, and the first superior molar of the right side, with an anterior proximal cavity, both large. On excavating, considerable sensitiveness of the dentine was found to exist, particularly immediately beneath the enamel, and very little elsewhere. The pulps were not affected by the decay. After the cavity in the left tooth was formed, it was very slightly sensitive; not sufficiently so to be unpleasant to the patient. No application was made to allay the sensitiveness. The tooth was well filled with gold. The cavity in the other tooth being excavated, was quite sensitive till the excavation was completed, especially round the border of the cavity. As the patient had good powers of endurance, it was not deemed necessary to make any application to destroy the sensitiveness; and so the cavity was filled, with considerable sensitiveness remaining, as above described. The introduction of the filling, however, gave no pain of consequence; it was well filled and finished in the usual manner. The first of these exhibited no sensitiveness for three or four weeks, but after the lapse of about that time, it became very sensitive to cold or heat, so that a very slight variation of temperature produced intense pain; the other was very sensitive to heat or cold from the first. So great was this difficulty, that both teeth were rendered almost useless in mastication. They continued in that condition, with unabated intensity, till the 13th of June following.

The patient was in good health, and constitutional treatment was not indicated. On the 13th June, I removed both fillings, and found the dentine immediately beneath the enamel in both teeth, very sensitive; the dentine elsewhere in the cavity was not sensitive. The cavities were excavated, till it was ascertained the sensitive portion could not be cut away. Chloride of zinc with chloroform was then applied; and in about one minute the sensitiveness was wholly destroyed. I then filled both teeth, and am now awaiting the result.

Was there any method of remedying the difficulty without removing the fillings?

Will the difficulty be liable to recur?

Why does the dentine become more sensitive in such cases after filling?

